# Clinical Manifestations of Leukocytoclastic Vasculitis, Treatment, and Outcome in Patients with Ulcerative Colitis: A Systematic Review of the Literature

**DOI:** 10.3390/jcm11030739

**Published:** 2022-01-29

**Authors:** Ivana Pantic, Djordje Jevtic, Charles W. Nordstrom, Cristian Madrid, Tamara Milovanovic, Igor Dumic

**Affiliations:** 1 Clinic of Gastroenterology and Hepatology, University Clinical Centre of Serbia, 11000 Belgrade, Serbia; ilic.ivana04@gmail.com (I.P.); tamara.alempijevic@med.bg.ac.rs (T.M.); 2 School of Medicine, University of Belgrade, 11000 Belgrade, Serbia; djordje965@gmail.com; 3 Department of Hospital Medicine, Mayo Clinic Health System, Eau Claire, WI 54702, USA; nordstrom.cw@mayo.edu (C.W.N.); Madrid.Cristian@mayo.edu (C.M.); 4 Mayo Clinic Alix School of Medicine, Rochester, MN 55905, USA

**Keywords:** leukocytoclastic vasculitis, ulcerative colitis, Crohn’s disease, vasculitis, hypersensitivity vasculitis

## Abstract

Leukocytoclastic vasculitis (LCV) is a rare extraintestinal manifestation (EIM) of ulcerative colitis (UC). Observations about its association with UC stem from case reports and small case series. Due to its rarity, more rigorous cross-sectional studies are scarce and difficult to conduct. The aim of this systematic review was to synthetize the knowledge on this association by reviewing published literature in the form of both case reports and case series; and report the findings according to the Preferred Reporting Items for Systematic Reviews and Meta-Analyses (PRISMA) guidelines. In contrast to LCV in Chron disease (CD), which occurs secondary to biologic therapies used for its treatment, LCV in UC is a true reactive skin manifestation. Both genders are equally affected. Palpable purpura (41%) and erythematous plaques (27%) are the most common clinical manifestations. In 41% of patients, the rash is painful, and the lower extremities are most commonly involved (73%). Systemic symptoms such as fever, arthralgias, fatigue, and malaise are seen in 60% of patients. Unlike previous reports, we found that LCV more commonly occurs after the UC diagnosis (59%), and 68% of patients have active intestinal disease at the time of LCV diagnosis. Antineutrophil cytoplasmic antibody (ANCA) is positive in 41% of patients, and 36% of patients have other EIMs present concomitantly with LCV. The majority of patients were treated with corticosteroids (77%), and two (10%) required colectomy to control UC and LCV symptoms. Aside from one patient who died from unrelated causes, all others survived with their rash typically resolving without scarring (82%).

## 1. Introduction

Vasculitis represents inflammation of the blood vessel wall, further classified by the size of the blood vessels involved [1]. Small vessel vasculitides affect arterioles, capillary, and postcapillary venules. Leukocytoclastic vasculitis (LCV) is a small vessel immune complex mediated vasculitis of the skin [1,2]. It is most frequently associated with infections, medications, cancer, and autoimmune diseases. Ulcerative colitis (UC) is one of the two main subtypes of inflammatory bowel disease (IBD). Differing from Crohn’s disease (CD), where pathologic changes are transmural and patchy, UC lesions are continuous and restricted to the mucosal and submucosal layers [3]. Our understanding of the association between vasculitis and IBD is limited, and is derived from case reports, case series, and small observational studies. Despite the limited data; however, it appears that these entities do occur more frequently than what would be expected to arise by chance [4]. LCV is considered to be a reactive cutaneous manifestation of IBD [5,6,7], but can also develop secondary to the medications used for IBD treatment [8].

The objective of this systematic review is to synthetize data on LCV in patients with UC by reviewing both case reports and case series and reporting on the patients’ characteristics, presentations, treatment, and outcome. LCV in CD has been already described elsewhere [8].

## 2. Materials and Methods

A systematic review of the literature was conducted by searching the PubMed/MEDLINE database starting from January 2000 to 15 November 2021 to identify published cases and case series of patients with UC and LCV, in accordance with Preferred Reporting Items for Systematic Reviews and Meta-Analyses (PRISMA) guidelines. Search terms included “ulcerative colitis and leukocytoclastic vasculitis”, “ulcerative colitis and small vessel vasculitis”, “ulcerative colitis and hypersensitivity vasculitis”, “ulcerative colitis and hypersensitivity angiitis”, “ulcerative colitis and leukocytoclastic angiitis”, “inflammatory bowel disease and leukocytoclastic vasculitis”, “inflammatory bowel disease and small vessel vasculitis”, “inflammatory bowel disease and hypersensitivity vasculitis”, “inflammatory bowel disease and hypersensitivity angitis”, and “inflammatory bowel disease and leukocytoclastic angitis”.

**Scheme 1:**
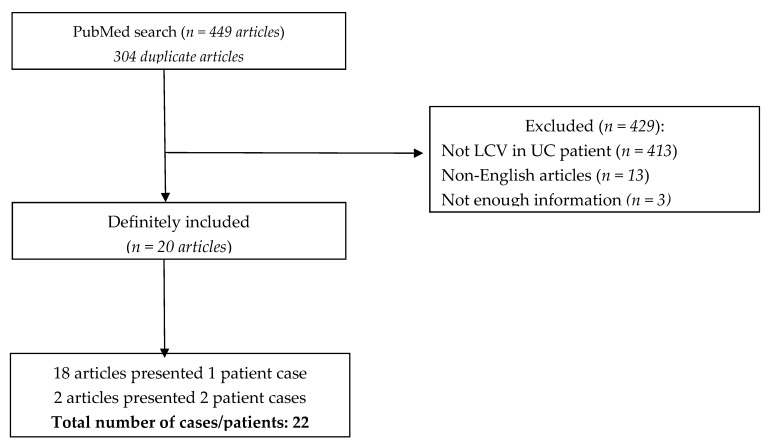
PRISMA flow chart: In order to be included, all cases reviewed had to report biopsy of the skin and colonic mucosa, with pathohistological descriptions consistent with the diagnosis of LCV and UC. Cases in which skin biopsy specimen was missing were included only if clinical presentation and clinical course were highly suggestive of the diagnosis of LCV in UC, and all co-authors independently agreed upon that matter.

Two authors blindly and independently searched and selected the cases (I.P. and D.J.). Only articles published in journals indexed by PubMed were taken into account. A total of 449 articles were identified, of which 304 were duplicates. References to the selected articles were further searched with no additional articles identified. The selection process resulted in the inclusion of a total of 20 articles for this study [9,10,11,12,13,14,15,16,17,18,19,20,21,22,23,24,25,26,27,28] and a total of 22 cases included in this review. The PRISMA flow-chart is illustrated below (Scheme 1).

An Excel spreadsheet was created to track the following data: age, sex, comorbidities, probable etiology of LCV (UC itself vs. precipitated by administered medications), IBD therapy (including colectomy) and location, IBD activity at the time of rash occurrence, rash location, description, symptoms and recurrence, rash occurrence timeline, presence of scarring, skin biopsy (if applicable), presence of other extraintestinal IBD manifestations, laboratory parameters of inflammation (erythrocyte sedimentation rate [ESR] and C-reactive protein [CRP] values), presence of anti-neutrophil cytoplasmatic antibodies (ANCA) at the time of rash occurrence, LCV treatment modalities, misdiagnosis of the rash, and patient outcome (defined as recovered or death).

## 3. Results

### 3.1. Demographic Characteristics and Co-Morbidities

The median age of patients described in this review was 18 years (range 2–79), with no predominance regarding sex (males: *n* = 11, 50%). Half of the patients were pediatric cases (*n* = 11, 50%). The majority of patients had no co-morbidities (*n* = 20, 90.91%) (Table 1). In one patient, coronary artery disease, diabetes mellitus, and hypertension were reported, while in another case, only diabetes mellitus was noted.

### 3.2. LCV Etiology and Clinical Presentation

In the vast majority of reviewed patients, the LCV etiology was UC irrespective of disease activity (*n* = 21, 95.45%). In only one patient (4.55%) was the therapeutic agent (infliximab) identified as the precipitating factor for LCV.

Clinical presentation of LCV is heterogenous. Reported skin changes vary from petechial rash to necrotizing ulcers. Nevertheless, the most commonly reported efflorescences were purpura (*n* = 9), erythematous plaques (*n* = 6), and serous and/or hemorrhagic bullae (*n* = 5) (40.91%, 27.27%, and, 22.73%, respectively). In two cases, erythematous plaques were specified as shapes described as “annular” and “target-like”. Rash was most commonly bilateral and manifested on the lower extremities (*n* = 16, 72.73%); however, the upper extremities (*n* = 9) and trunk (*n* = 7) were also frequently involved (40.91% and 31.82%, respectively). Unusual locations such as intravenous catheter insertion site, and palms and soles were reported in one patient each (4.55%). Pain was the most common accompanying rash symptom, occurring in nine patients (40.91%). Rash was recurrent in nine patients (40.91%), and left scars in four patients (18.18%). During the rash occurrence, arthralgias were reported in ten patients, fever in two, and fatigue in three (45.45%, 9.09%, and 13.64%, respectively). LCV preceded UC diagnosis in only two patients (9.09%). In seven patients (31.82%), LCV occurred concurrently with UC diagnosis/symptoms, and occurred after the diagnosis of UC was established in thirteen (59.09%) patients. Misdiagnosis was reported in five patients (22.73%) comprising Henoch–Schönlein purpura in three patients (60%), and allergic reaction in two (40%). One patient (4.55%) died from unrelated causes. Data regarding LCV etiology and clinical presentation are presented in detail in Table 1.

### 3.3. UC Clinical Characteristic

In patients described in this review, pancolitis was the most common form of UC (*n* = 9, 40.91%). Active disease (either disease flare or first manifestation) at the time of rash occurrence was detected in fifteen patients (68.18%). In four patients (18.18%), UC was in a remission state when rash appeared. Other extra-intestinal manifestations were reported in eight patients (36.36%) and included pyoderma gangrenosum, erythema nodosum, and primary sclerosing cholangitis in two patients each (25%) and episcleritis and psoriasis in one patient each (12.5%). Data regarding UC clinical characteristics are presented in detail in Table 1.

### 3.4. Laboratory Parameters during LCV Occurrence

Median values of erythrocyte sedimentation rate (ESR) and C-reactive protein (CRP) at the time of LCV occurrence were 49.5 mm/h (range 2–84) and 58.5 mg/L (3–325.3), respectively. Five patients (22.73%) were positive for perinuclear anti-neutrophil cytoplasmic antibodies (p-ANCA), four (18.18%) were positive for anti-neutrophil cytoplasmic antibodies (c-ANCA), while in 10 patients (45.45%), no ANCA was detected. In three patients (13.64%), data regarding ANCA positivity was missing. Data regarding laboratory parameters during LCV occurrence are presented in Table 1.

### 3.5. Treatment Modalities Administered for LCV in Patients with UC

Most patients were treated with corticosteroids (*n* = 17, 77.27%). Among thee corticosteroids, prednisone or prednisolone were administered in more than 2/3 of patients (76.47%). Aminosalicylates and steroid-sparing immunosuppressants were administered in nine (40.91%), and eight (36.36%) patients, respectively. Biologic agents were given in five cases (22.73%). Due to the inability to control UC disease course, colectomy was performed in two patients (9.09%) (Table 2).

## 4. Discussion

Extraintestinal manifestations (EIMs) in IBD are common, occurring in up to 40% of patients [6,29]. They are more common in patients with CD than in those with UC. In the Swiss Inflammatory Bowel Disease Cohort (SIBDC), 31% of patients with UC exhibited one of the EIMs. Skin, joints (arthritis), eye (uveitis), and biliary tract (primary sclerosing cholangitis) are the most common sites of EIMs in patients with UC. Based on pathogenesis, cutaneous manifestations of IBD are categorized as one of the following: reactive cutaneous manifestations, granulomatous cutaneous lesions, dermatosis associated with IBD, or secondary cutaneous manifestation due to complication of IBD treatment [5]. LCV is considered to be a reactive cutaneous manifestation of IBD; however, it may also be secondary to the medication used for its treatment [8,12].

The association of IBD and vasculitis is complex, and the exact incidence is unknown. One of the reasons why it is so difficult to determine the precise interrelationship is the fact that vasculitis (and particularly LCV) is an exceedingly rare extraintestinal IBD manifestation. For example, a recent study from several large tertiary care hospitals in North America spanning several years described only 32 unique patients with IBD and vasculitis [4]. In this study, only five patients had isolated cutaneous vasculitis (LCV), of which only two occurred in patients with UC. This illustrates the rarity of co-occurrence of these entities [4].

The pathogenesis of IBD and associated EIMs is complex and multifactorial consisting of the individual’s genetic predisposition and environmental factors [30]. T cells located in the intestinal mucosa are a crucial element of our immune system in maintaining intestinal equilibrium between bowel mucosa, microorganisms (bacteria and fungi), and host immune response [5,30,31,32]. A dysregulated immune response by these T cells against the intestinal microbiome has been implicated as the initial step in a cascade of events, ultimately leading to chronic inflammation, abnormal cytokine production, and development of IBD and EIMs [5]. In addition to cell mediated pathways by Th1, and humoral immunity mediated by Th2, another T helper cell, Th17, has been associated with various autoimmune and inflammatory diseases including IBD and its EIMs [33,34]. IL-23 induced by IL-17 (which is in turn produced by Th17) has emerged as an important factor in the pathogenesis of IBD and its cutaneous manifestations. Hence, monoclonal antibodies directed against these interleukins (e.g., ustekinumab, directed against IL-23) have been found to be very effective in the treatment of these difficult to treat conditions.

LCV as a reactive cutaneous manifestation of UC is thought to occur due to dysregulation of the immune system in genetically predisposed individuals with UC. Inflamed intestinal mucosa and “leaky gut” lead to unregulated penetration of microorganisms and their antigens through the mucosal barrier, which triggers an abnormal immune reaction [35]. The immune system, being unable to discriminate between epitopes on microbial antigens and epitopes of normal tissue (synovia, skin, blood vessels, eye), attacks both. In LCV, immune complexes are deposited in postcapillary venules, and the destruction of their walls leads to blood extravasation and rash development. In fact, it has been shown that people with more extensive IBD and more active intestinal disease are at an increased risk of developing these EIMs [29,36]. A common pathogenesis of these conditions is further supported by the fact that they tend to occur together, and the occurrence of one EIM increases the risk for the development of subsequent EIMs [29]. An autoimmune reaction against tropomyosin-related peptide is thought to be the main step in the pathogenesis of EIMs [37]. Given the fact that this isoform of tropomyosin is abundant in ciliary epithelium of the eye, keratinocytes, epithelium of the biliary tree, and chondrocytes, it is not surprising that these organs are the most commonly affected extraintestinal structures in patients with IBD [29,37]. A genetic component for the development of these manifestations has also been identified. For example, patients with UC and the HLA-B8 and HLA-DR 3 phenotypes are 10 times more likely to develop PSC [29,38]. People with UC and the HLA-DR 103 phenotype are more likely to develop skin manifestations [29,38]. The gene TRAF3IP2 is associated with the development of both IBD phenotypes [39]. Concordance between siblings of more than 80% in the development of EIMs further supports the fact that genetic makeup is one of the most important determinants of who will develop EIMs [40].

While some of the previous studies found that females more commonly developed EIMs [38], in this systematic review, we found that LCV occurs equally in both genders. Previously identified risk factors for the development of skin manifestations in patients with IBD are female gender, family history of IBD, and younger age at IBD diagnosis. A recent study by Vavricka, however, demonstrated these factors to be predictive of EIM development in patients with CD, but not UC [29]. Of note, this study did not include patients with LCV specifically.

The majority of the patients in this review developed LCV as reactive manifestation of UC (95%), and only one patient (5%) developed LCV as a consequence of anti-tumor necrosis factor (TNF-alfa) therapy [12]. This finding is strikingly different from patients with LCV and CD, where LCV attributed to IBD therapy (most commonly infliximab and adalimumab) was found in 60% of patients [8,41]. In one of the cases, a streptococcal infection preceded both rash occurrence and UC symptoms. Therefore, in this specific patient, LCV may have been precipitated by either prior infection, UC, or potentially both [27]. Table 3 demonstrates differences between LCV in CD and UC.

Palpable purpura was the most common clinical finding on skin examination in 40% of patients, followed by erythematous plaques found in 27%. Other less common presentations are illustrated in Table 1. The rash was painful in 40% of patients, pruritic in 30%, and in 22% was initially misdiagnosed as an allergic dermatosis or Henoch–Schönlein purpura (HSP). Another common mimicker for LCV is cellulitis, however, it should be noted that in the majority of cases, LCV occurred bilaterally, whereas cellulitis was almost always unilateral. Unlike the other more common cutaneous manifestations of IBD (e.g., erythema nodosum and pyoderma gangrenosum), which are clinical diagnoses, LCV diagnosis always requires a skin biopsy.

The temporal relationship between the occurrence of skin manifestations and IBD is also complex, and not completely understood. Dermatologic manifestations may precede the development of gastrointestinal symptoms, occur in parallel, or develop later in the course of UC (either during exacerbation or quiescence). It has previously been thought that LCV is more common in patients with UC than in CD, and precedes the onset of bowel disease [5]. We described 22 patients in this review, meanwhile, a previous paper described 14 patients of LCV in CD [8]. It is evident that there are more publications on LCV in UC. Contrary to prior reports [5,26], we found that in the majority of patients (59%), LCV occurred after the diagnosis of UC. The majority of the reviewed patients (68%) developed LCV during an active UC episode, and not during remission. These findings replicate those from the SIBDC, which found that skin manifestations (and other EIMs) are more likely to occur during the active phase of IBD [29,38]. Some EIMs follow the disease activity (e.g., erythema nodosum), while others (e.g., pyoderma gangrenosum) occur independently of the clinical course in UC [5,29,38]. In 68% of patients described in this review, LCV occurred during an active phase of UC, while in 18%, it occurred during UC remission. In 36% of patients, LCV co-occurred with another EIM, and it has been previously described that the development of one EIM increases the risk for other EIM development [29].

LCV may occur as an isolated dermatologic vasculitis, or it may be a part of systemic vasculitis, where the skin is among many organs involved; this can be challenging from differential diagnosis standpoint. Unfortunately, in many of the reported cases, the distinction between isolated vs. systemic had not been clearly documented. While only 10% of patients in this review had concurrent fever with rash, 45% had arthralgias. Our review replicates findings from previous studies reporting musculoskeletal complaints, especially joint pain, to be a common complaint in patients with EIM of IBD [6,29,38]. The small vessel vasculitis, in addition to skin, may affect the bowels, presenting as abdominal pain, gastrointestinal bleeding, and/or fever. These symptoms and signs are indistinguishable from UC flare or opportunistic infections. In these situations, the practicing clinician must first rule out infection (which warrants targeted antimicrobial treatment) and evaluate UC activity. Therefore, even if patients present with isolated skin lesions without apparent involvement of other organs, a systematic diagnostic evaluation should still be conducted. Inflammatory markers such as CRP and ESR are frequently elevated in infections, IBD flares, and LCV, hence their diagnostic utility is limited in differentiating between these entities. Once infection is ruled out, then treatment with immunosuppressive medication should be started.

Treatment for LCV depends on the severity of the vasculitis and UC. Mild cases usually respond well to systemic steroid treatment. More severe forms, however, may require application of several different immunosuppressive agents (similar to a UC exacerbation). In the majority of the reviewed cases, systemic corticosteroids were recommended (parenteral or peroral), and our findings also support this. In our reviewed cases, 77% of patients were treated with systemic steroids, with the LCV rash usually subsiding quickly. In 80% of cases, resolution was without scarring, unlike pyoderma gangrenosum, which usually leaves dramatic dermatologic sequela. Should LCV occur with UC exacerbation, both entities usually respond simultaneously to systemic steroid therapy. Interestingly, in a few cases, patients did not respond to steroid therapy, but had a favorable outcome with aminosalicylates [14,19,20,21,22,26,28]. In challenging cases, when all treatment modalities had failed to control the UC exacerbation and LCV, colectomy was performed as a last resort, and resulted in improvement in the rash [14,18].

This systematic review has a few limitations that are inherent to the methodology of the review. First, we only had selected cases from the journals that are indexed in PubMed. While doing so, we wanted to eliminate low quality publications. However, we might have omitted some high quality reports by doing so. Second, we only included articles in English. Finally, publication selection bias is a well-known limitation of these types of reviews, which also pertains to our review.

## 5. Conclusions

To summarize, this is the first systematic review of clinical characteristics, treatment, and outcome of LCV in patients with UC. We described 22 patients, and found that unlike previously thought, LCV usually occurs after the diagnosis of UC is established, and rarely does it precede gastrointestinal symptoms. Unlike in CD, where secondary LCV due to biologic therapy is common, LCV in UC is a true reactive skin manifestation. Treatment of IBD in all cases resulted in LCV remission, however, in two cases, colectomy was necessary to control the symptoms of both UC and LCV.

## Figures and Tables

**Table 1:** The epidemiology, demographics, clinical presentation, diagnostic findings, and outcome in LCV in UC cases.

Demographic Characteristics			
Gender	*n* (%)	Median age (years)	Age range (years)
Female	11 (50%)	12	(2–66)
Male	11 (50%)	28	(5–79)
Total	22 (100%)	18	(2–79)
**Co-morbidities**			
Yes	2 (9.09%)		
No	20 (90.91%)		
**LCV etiology**			
UC	21 (95.45%)		
Therapy	1 (4.55%)		
**Clinical presentation**			
**Rash description**			
Purpura	9 (40.91%)		
Erythematous plaques	6 (27.27%)		
Serous and/or hemorrhagic bullae	5 (22.73%)		
Erythematous macules	4 (18.18%)		
Purpuric plaques	3 (13.64%)		
Petechiae	2 (9.09%)		
Ecchymosis	1 (4.55%)		
Hyperpigmented maculae	1 (4.55%)		
Subcutaneous nodules	1 (4.55%)		
Necrotizing ulcers	1 (4.55%)		
**Rash location**			
Lower extremities	16 (72.73%)		
Upper extremities	9 (40.91%)		
Trunk	7 (31.82%)		
Buttocks	5 (22.73%)		
Loins	2 (9.09%)		
Other (i.v. catheter insertion site, palms, soles, neck)	4 (18.18%)		
**Rash symptoms**			
Pain	9 (40.91%)		
Pruritus	6 (27.27%)		
Dysesthesia	1 (4.55%)		
ND	8 (36.36%)		
**Rash recurrence**			
Yes	9 (40.91%)		
No	13 (59.09%)		
**Scarring**			
Yes	4 (18.18%)		
No	18 (81.82%)		
**Systemic symptoms**			
Arthralgia	10 (45.45%)		
Fever	2 (9.09%)		
Fatigue	3 (13.64%)		
None	9 (40.91%)		
**Timing of rash onset**			
Before UC diagnosis/symptoms	2 (9.09%)		
Concurrent with UC	7 (31.82%)		
After UC diagnosis	13 (59.09%)		
**UC location**			
Pancolitis	9 (40.91%)		
Proctosigmoiditis	1 (4.55%)		
Proctitis	1 (4.55%)		
Left-sided with rectal sparing Left-sided ND	1 (4.55%) 1 (4.55%) 9 (40.91%)		
**UC activity at the time of rash occurrence**			
Active	15 (68.18%)		
Remission	4 (18.18%)		
Occurred before UC diagnosis	2 (9.09%)		
ND	1 (4.55%)		
**Other extraintestinal manifestations**			
Yes	8 (36.36%)		
Psoriasis	1 (12.5%)		
Erythema nodosum	2 (25%)		
Pyoderma gangrenosum	2 (25%)		
Episcleritis	1 (12.5%)		
Primary sclerosing cholangitis	2 (25%)		
No	14 (63.64%)		
Erythrocyte sedimentation rate, mm/h (median, range) *	49.5 (2–84)		
C-reactive protein, mg/L (median, range) **	58.5 (3–325.3)		
**Serology**			
p-ANCA	5 (22.73%)		
c-ANCA	4 (18.18%)		
None	10 (45.45%)		
ND	3 (13.64%)		
**Misdiagnosis**			
Yes	5 (22.73%)		
Allergic reaction	2 (40%)		
Henoch–Schonlein purpura	3 (60%)		
No	17 (77.27%)		
**Outcome**			
Recovered	21 (95.45%)		
Death	1 (4.55%)		

* Data missing or exact value not specified in 12 cases. ** Data missing or exact value not specified in 10 cases. LCV—leucocytoclastic vasculitis; UC—ulcerative colitis; ND—no data; p-ANCA—perinuclear anti-neutrophil cytoplasmic antibodies; c-ANCA—anti-neutrophil cytoplasmic antibodies.

**Table 2:** Therapeutic options for LCV administered to the patients with UC described in this review.

Therapeutic Agent Groups	*n* (%)
Corticosteroids *	**17 (77.27%)**
Prednisone/prednisolone	13/17 (76.47%)
Hydrocortisone	2/17 (11.76%)
Methylprednisolone	2/17 (11.76%)
Deflazacort	1/17 (5.88%)
Unspecified	2/17 (11.76%)
**Aminosalycilates**	**9 (40.91%)**
Mesalamine	4/9 (44.44%)
Sulfasalazine	5/9 (55.56%)
Steroid-sparing immunosupressants *	**8 (36.36%)**
Cyclosporine	3/8 (37.5%)
Azathioprine	3/8 (37.5%)
6-mercaptopurine	2/8 (25%)
Methotrexate	1/8 (12.5)
Mycophenolate mofetil	1/8 (12.5%)
Biologic agents *	**5 (22.73%)**
Infliximab	2/5 (40%)
Adalimumab	2/5 (40%)
Ustekinumab	1/5 (20%)
Rituximab	1/5 (20%)
**Colectomy**	**2 (9.09%)**
**Topical therapy**	**2 (9.09%)**
**Other (colchicine, dapson, hydroxychloroquine)**	**3 (13.64%)**

* Some patients received several different drugs belonging to the same group due to the inability to achieve adequate therapeutic response or development of allergic reaction to the drug.

**Table 3:** Comparison of LCV in UC and CD.

Leukocytoclastic Vasculitis	
Ulcerative Colitis	Crohn’s Disease
1. Can precede UC diagnosis but most commonly occurs after UC has been diagnosed	1. No documented cases where LCV precedes CD diagnosis
2. True reactive skin manifestation, rarely secondary to medications	2. Most of the cases are secondary to biologic therapy used in the treatment of CD
3. Requires treatment and there is no documentation about spontaneous resolution	3. Can resolve spontaneously without specific therapy

## Data Availability

All data are publicly available on the PubMed database.
